# Supplementary material for: The Neoangiogenic Transcriptomic Signature Impacts Hepatocellular Carcinoma Prognosis and Can Be Triggered by Transarterial Chemoembolization Treatment
Source: Cancers (Basel). 2024 Oct 21;16(20):3549. doi: 10.3390/cancers16203549 (PMC11505901; doi:10.3390/cancers16203549)
Supplement: Supplementary file 1 [file cancers-16-03549-s001.zip › cancers-3201555-supplementary.pdf]

The neoangiogenic transcriptomic signature affects HCC prognosis and is triggered by TACE treatment

Rosina Maria Critelli, Federico Casari, Alberto Borghi, Grazia Serino, Cristian Caporali, Paolo Magistri, Annarita Pecchi, Endrit Shahini, Fabiola Milosa, Lorenza Di Marco, Alessandra Pivetti, Simone Lasagni, Filippo Schepis, Nicola de Maria, Francesco Dituri, Maria-Luz Martinez-Chantar, Fabrizio Di Benedetto, Gianluigi Giannelli, Erica Villa

Table of contents

Supplementary Figure S1 page 2
Supplementary Figure S2

Supplementary tables

Supplementary Table S1 page 3
Supplementary Table S2 page 5

Supplementary Method page 7

Supplementary Figure S1

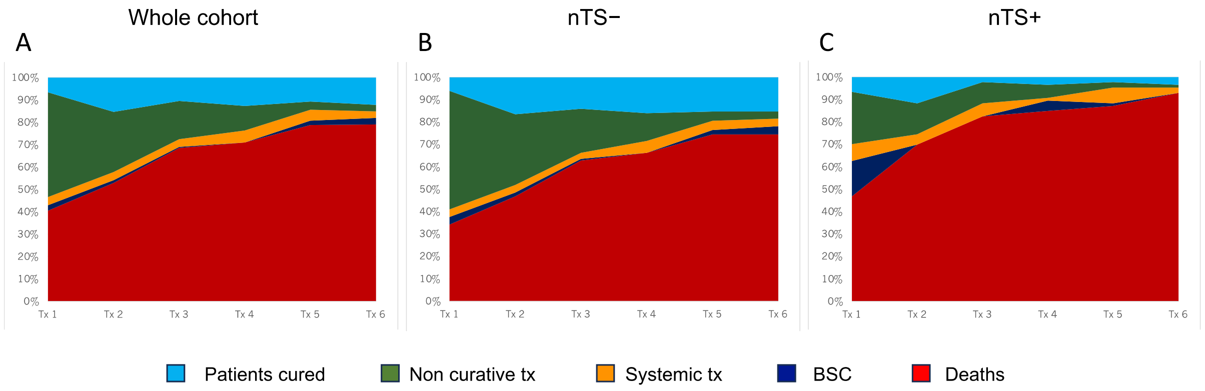

Outcome of the sequential treatments performed in the derivation cohort of patients with HCC. At each time point, the cumulative percentage of patients in each category is indicated. Data are reported for the whole cohort (A) and for the nTS– (B) and nTS+ (C) cohort. Survival at the end of the observation was significantly lower in the nTS+ cohort (p<0.001, log rank test).

Supplementary Figure S2

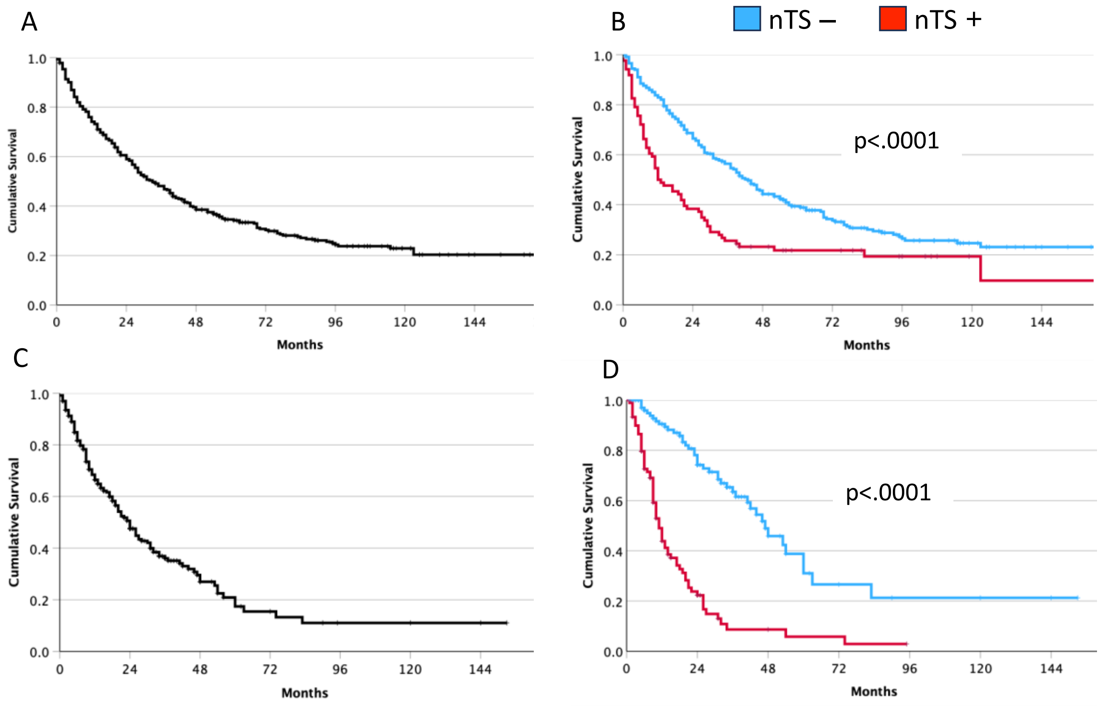

Kaplan–Meier analysis for survival of patients belonging to the derivation (A,B) and validation (C,D) cohorts. In A and C global survival is reported. In B and D, survival is stratified by presence or absence of the neoangiogenic transcriptomic signature (nTS). Presence of the latter was associated with a significantly worse survival.

Supplementary Table S1 - Characteristics at enrolment of HCC patients of derivation and validation cohorts, stratified by neoangiogenic Transcriptomic Signature (nTS) (E-S grade: Edmondson-Steiner grade; Plt: Platelets; PVT: Portal Vein Thrombosis)

	Derivation cohort				Validation cohort			
**Variable**	**All cases (n = 328)(%)**	**nTS- (n = 242)(%)**	**p**	**nTS+ (n = 86)(%)**	**All cases (n= 256)(%)**	**nTS- (n=176)(%)**	**p**	**nTS+ (n=80)(%)**
Males	259 (79.0)	193 (79.5%)		66 (76.7%)	205 (80.1)	142 (81.1)		61 (77.6)
Age (y)	65.1±11.2	65.5±10.8		64.7±12.2		61.1±10.7		60.0±11.4
Etiology viral/non-viral	213/115 (64.9/35.1)	157/85 (64.9%/35.1%)		56/30 (65.1%/35.1 %)	170/74 (69.7/30.3)	109/67 (61.9%/38.1 %)		52/28 (65.0%/35.0 %)
Plt count (10^3/mmc)	127.9±74.0	124.8±73.2		138.4±75.6	81.5±42.2	67.3±21.3		97.4±55.3
Bilirubin (mg/dl)	1.5±2.1	1.5±2.3		1.6±1.7	2.3±2.1	1.9±2.3		1.8±1.7
Albumin (g/l)	3.5±0.59	3.6±0.5		3.5±0.6	3.6±0.4	3.4±0.4		3.4±0.3
INR	1.2±0.3	1.2±0.2		1.2±0.3	1.3±0.3	1.4±0.2		1.3±0.3
Creatinine (mg/dl)	.88±.30	0.89±0.3		0.86±0.3	0.9±0.2	0.90±0.4		0.87±0.3
AFP (ng/ml)	1740±8900	954±6209	p<.05	3896±1300	1232±7110	655±5900	p<.05	1938±8624
Child-Pugh A/B/C	227/85/15 (69.4, 26.0, 4.6)	169/63/10 (69.8/26.0/4.1)		58/22/5 (68.2%/25.9, 5.9%)	138/98/20 (53.8/38.2/7.8)	95/68/13 (53.9/38.8/7.)		54/20/6 (67.5/25.0/7.5)
MELD score	10.5±3.1	10.7±3.1		9.91±3.3	11.0±5.0	10.0±4.3		9.7±4.3
BCLC A/B/C/D	186/82/45/15 (57.0/25.0/13.7/4.6)	149/56/29/8 (61.6/23.1/12.0/3.3)		34/28/18/6 (39.5/32.5/21.0/7.0)	146/68/32/10 (52.0/26.9/12.5/3.9)	99/46/21/10 (56.2/26.1/12.0/5.7)		32/26/17/5 (40.0/32.5/21.2/6.3)
HCC nodules<br>1<br>2<br>3<br>multifocal	192 (58.5)<br>65 (19.8)<br>10 (3.0)<br>61 (18.6)	152 (62.8%)<br>51 (21.1%)<br>5 (2.1%)<br>34 (14.0%)	p<0.001	40 (46.5)<br>14 (16.3%)<br>5 (5.8%)<br>27 (31.4%)	146 (57.0)<br>53 (21.0)<br>4 (1.5)<br>53 (20.7)	115 (65.3)<br>39 (22.2)<br>3 (1.7)<br>19 (11.0)		35 (43.8)<br>14 (17.5)<br>1 (1.2)<br>30 (37.5)
E-S grade								
I	108 (33.0)	94 (39.0)		14 (16.3)	72 (28.1)	66 (37.5)		12 (15.0)
II	121 (37.0)	84 (34.9)	p<0.001	37 (43.9)	100 (39.0)	64 (36.4)	p<0.001	36 (45.0)

III	83 (25.4)	57 (23.7)		26 (30.2)	71 (27.7)	41 (23.3)		22 (27.5)
IV	15 (4.6)	6 (2.5)		9 (10.5)	13 (5.0)	5 (2.8)		10 (12.5)
PVT	46 (14.0)	29 (12.0)		17 (19.8)	38 (15.0)	24 (13.6)		14 (17.5)

Supplementary Table S2

List of consecutive therapeutic measures performed in the derivation cohort of patients with HCC after first diagnosis.

	1st			2nd			3rd			4th			5th			6th		
**Treatment**	**All**	**TS-**	**TS+**	**All**	**TS-**	**TS+**	**All**	**TS-**	**TS+**	**All**	**TS-**	**TS+**	**All**	**TS-**	**TS+**	**All**	**TS-**	**TS+**
**n.**	**328**	**242**	**86**	**175**	**146**	**29**	**101**	**86**	**15**	**55**	**44**	**11**	**33**	**26**	**7**	**6**	**5**	**1**
Best supportive care (n, %)	52 (15.9)	34 (14.0)	18 (20.9)	10 (5.7)	10 (6.8)	0	3 (3.0)	2 (2.3)	1 (6.7)	-	-	-	2 (6.0)	1 (3.8)	1 (14.2)	0	0	0
Curative treatment (n, %)*	117 (35.7)	90 (37.1)	26 (30.2)	32 (18.2)	27 (18.4)	5 (17.4)	17 (16.8)	15 (17.4)	2 (13.3)	5 (9.0)	5 (11.3)	1 (9.0)	1 (3.0)	0	1 (14.2)	0	0	0
Resection	58	37	21	9	7	2	16	14	2	1	1	0	0	0	0	0	0	0
RF	59	53°	6°	23	20	3	1	1	0	4	4	0	1	0	1	0	0	0
TACE (n, %)	105 (32.0)	88** (36.3)	17** (19.7)	86 (41.1)	74 (50.6)	12 (41.3)	43 (42.6)	37 (43.0)	6 (40.0)	27 (49.0)	22 (50.0)	5 (45.4)	14 (42.4)	13 (50.0)	1 (14.2)	2 (33.3)	2 (20.0)	0
Liver transplant (n, %)§	3 (0.9)	2 (0.83)	1 (1.1)	11 (6.2)	10 (6.8)	1 (3.4)	15 (14.8)	15 (17.4)	0	9 (16.3)	8 (18.1)	1 (2.2)	3 (9.0)	3 (11.5)	0	1 (16.6)	0	1
Systemic treatment (n, %)	51 (15.8)	28*** (11.9)	23*** (26.7)	36 (20.5)	25 (17.1)	11 (37.9)	23 (22.8)	17 (19.8)	6 (40.0)	14 (25.4)	9 (20.4)	4 (36.3)	13 (39.3)	9 (34.6)	4 (57.1)	3 (50.0)	3 (60.0)	0

°, °° p<0.0001
*, ** , *** , § p<0.05
***Patients who maintained a therapeutic response for more than 1 year before a possible subsequent treatment were classified as cured.

Supplemental Methods

(according to Villa et al. Gut doi.org/10.1136/gutjnl-2014-308483)

Definition of the neoangiogenic transcriptomic signature

Analysis of gene expression - Total RNA was isolated from non-tumor (NT) and tumor (T) liver tissues using Trizol (Invitrogen, Carlsbad, CA, USA) according to the manufacturer's instructions. The quality and quantity of the RNA samples were assessed using an Agilent Model 2100 Bioanalyzer (Agilent Technologies, Palo Alto, CA, USA) and an ND-1000 Spectrophotometer (NanoDrop Technologies, Wilmington, DE, USA), respectively. RNA was processed using 4x44K whole genome oligonucleotide-based gene expression microarrays (Agilent Technologies, Palo Alto, CA; Genomics Service Department of Miltenyi Biotec GmbH Bergisch Gladbach, Germany). Labeling and hybridization procedures were performed according to the instructions provided by Agilent using the Quick Amp Labeling Kit and the One Color Microarray-Based Gene Expression Analysis Protocol.
RNA integrity number (RIN) values ranged from 6.0 to 9 for all samples. Briefly, in the first step, 500 ng of total RNA was converted into cDNA using a T7 promoter primer. In the second step, the cDNA was converted into cRNA and labeled with Cy3-CTP. After purification, labeled cRNAs were hybridized to Agilent Whole Human Genome Oligo Microarrays 4x44K using the manufacturer's reagents and protocols. After washing, fluorescent signals of the hybridized Agilent Microarrays were detected using Agilent's Microarray Scanner System (Agilent Technologies). Feature extraction software provided by Agilent was used to quantify the intensity of fluorescent images and to normalize the results using a linear lowess method according to the manufacturer. All data were imported into Resolver software (Rosetta Biosoftware, Kirkland, WA) for database management, quality control, and analysis. Up-regulated and down-regulated genes were identified for the first doubling time quartile (e.g., fast-growing tumors) versus the other three quartiles. Only genes with an uncorrected p-value less than 0.01 and a 2-fold expression difference were selected. Gene expression data are available at the Gene Expression Omnibus website (www.ncbi.nlm.nih.gov/geo) under accession number: GSE54236.
Discriminatory gene analysis (DGA - To determine if there were genes differentially expressed between the fastest and slowest quartiles of all tumor samples, a discriminatory gene analysis (DGA) was performed. Each tumor sample was individually compared to the combined group of non-tumor samples. This approach identified genes that deviated from normality in multiple tumor samples, but not necessarily in all of them. These data were then used to distinguish between the fastest-growing and slowest-growing quartiles of the tumor samples. For the detection of genes that discriminate between fast and slow growing tumors, an in between groups t- test between the fastest and slowest quartiles has been performed. A list of 243 discriminatory genes, sorted by increasing "fast vs. slow" ratio, was identified. The biological relations were subsequently elucidated in a biological pathway analysis.
Functional Annotation and Biological Pathway Analysis - The functional annotation and all subsequent data analyses were performed by the bioinformatics service of Miltenyi Biotec (Genomics Service Department of Miltenyi Biotec GmbH Bergisch Gladbach, Germany). The most discriminatory genes were angiopoietin-2 (ANGPT2) and several genes encoding cell surface receptors and extracellular matrix proteins. This procedure identified groups of significantly regulated genes that are linked in terms of their importance in specific biological functions (e.g. angiogenesis). Five genes of the list are involved in angiogenesis (ANGPT2, ESM1, NETO2, NR4A1, DLL4). In addition to the functional annotation procedure, the gene sets were subjected to a manually curated biological pathway analysis. Each gene set was separately tested for significant enrichment of biological annotations and terms using Tree Ranker software (Miltenyi Biotec GmbH). This enrichment analysis aimed to identify Gene Ontology (GO) categories that might be enriched by the up- or down-regulated genes.

Risk Score Calculation - To determine whether gene expression profiles could predict the growth speed of HCC at presentation, the differentially upregulated and downregulated genes according to growth speed were ranked based on their predictive power using the univariate z-score, following the methodology of Lossos et al. (Prediction of survival in diffuse large-B-cell lymphoma based on the expression of six genes, N Engl J Med 2004;350:1828-37). A univariate Cox proportional-hazards analysis was performed with survival as the dependent variable. Genes with an absolute univariate z-score greater than 2.5 or less than -2.5 were then analyzed in a multivariate Cox proportional-hazards regression model, with survival as the dependent variable (p-values< 0.01 were considered statistically significant). The risk index was defined as a linear combination of the gene expression values for the top genes identified by the univariate Cox proportional-hazards regression model, weighted by their estimated regression coefficients (1.294 x Angpt2; 0.966 x DLL4; 0.726 x Neto2; 0.624 x NR4A1; 0.557 x ESM1). The 70th percentile cut point was chosen for its best discriminatory power.

MicroRNA

RNA Extraction and real-time PCR analysis

Mature microRNAs extracted from liver tumor and non-tumor tissue were reverse transcribed and amplified using the miRCURY LNATM Universal RT microRNA PCR system (EXIQON, Inc., Woburn, MA, USA). qRT-PCR was performed on a LightCycler 480 instrument (Roche, Mannheim, Germany) using miRCURY LNATM SYBR Green master mix (EXIQON) and Custom microRNA LNATM PCR primer set (EXIQON) in a 10 μl reaction volume, with 10 ng of complementary DNA (cDNA)/RNA concentration. The reactions were incubated in a 96-well plate at 95 °C for 10 minutes, followed by 45 cycles of 95 °C for 10 seconds and 60 °C for 1 minute. Each sample was analyzed in duplicate.

MicroRNA expression levels were measured using the Ct (threshold cycle) method. The 2-ΔΔCt method was employed for relative quantification of gene expression. The ΔCt was calculated by subtracting the Ct of U6 and RNAU5G from the Ct of the microRNA of interest. This approach allowed us to determine the fold changes in gene expression, normalized to two internal control genes and relative to the corresponding cirrhotic non-tumor liver tissue.

To cover possible pathways involved in tumor progression,, we selected a set of miRNA that are involved in promotion of proliferation capacity, migration ability, invasion ability, hypoxia-driven angiogenesis (miR-221-3p and miR-222-3p); in inhibition of apoptosis, growth, and upregulation in the hypoxic environment (mir-15b-5p; mir-16-5p); in inhibition of proliferation, invasion, and tumor growth (miR-30a-5p); in inhibition of autophagy (miR-30d-5p); in the negative regulation of cell proliferation (miR-145-5p); in cell cycle arrest, EMT and apoptosis (mir-122-5p); in the enhancement of hypoxia- induced migration and invasion of HCC cells (miR-210)
